# “CityQuest,” A Custom-Designed Serious Game, Enhances Spatial Memory Performance in Older Adults

**DOI:** 10.3389/fnagi.2022.806418

**Published:** 2022-03-08

**Authors:** Niamh A. Merriman, Eugenie Roudaia, Jan Ondřej, Matteo Romagnoli, Ivan Orvieto, Carol O’Sullivan, Fiona N. Newell

**Affiliations:** ^1^School of Psychology, Institute of Neuroscience, Trinity College Dublin, Dublin, Ireland; ^2^School of Computer Science and Statistics, Trinity College Dublin, Dublin, Ireland; ^3^Testaluna, Genoa, Italy

**Keywords:** aging, spatial navigation, video game, training intervention, balance control

## Abstract

Spatial cognition is known to decline with aging. However, little is known about whether training can reduce or eliminate age-related deficits in spatial memory. We investigated whether a custom-designed video game involving spatial navigation, obstacle avoidance, and balance control would improve spatial memory in older adults. Specifically, 56 healthy adults aged 65 to 84 years received 10 sessions of multicomponent video game training, based on a virtual cityscape, over 5 weeks. Participants were allocated to one of three training conditions: the main intervention, the “CityQuest” group (*n* = 19), and two control groups, spatial navigation without obstacle avoidance (“Spatial Navigation-only” group, *n* = 21) and obstacle avoidance without spatial navigation (“Obstacles-only” group, *n* = 15). Performance on object recognition, egocentric and allocentric spatial memory (incorporating direction judgment tasks and landmark location tasks, respectively), navigation strategy preference, and executive functioning was assessed in pre- and post-intervention sessions. The results showed an overall benefit on performance in a number of spatial memory measures and executive function for participants who received spatial navigation training, particularly the CityQuest group, who also showed significant improvement on the landmark location task. However, there was no evidence of a shift from egocentric to allocentric strategy preference. We conclude that spatial memory in healthy older participants is amenable to improvement with training over a short term. Moreover, technology based on age-appropriate, multicomponent video games may play a key role in cognitive training in older adults.

## Introduction

Spatial navigation, the ability to find our way between locations in an environment, is a complex cognitive function. To navigate successfully, an individual must recognize and remember salient landmarks, their relative locations, and the directions of previously taken routes. Two separate spatial strategies are thought to support these processes. First, an egocentric strategy involves the encoding of information related to the spatiotemporal sequence of environmental features relative to oneself, such as landmarks, and the sequence of movements necessary to get from one landmark to the next (Hartley et al., 2003; Wolbers et al., 2004). Second, an allocentric spatial strategy involves a more global representation or “cognitive map” of the environment where landmark locations are identified by their spatial relationship to one another (Tolman, 1948; O’Keefe and Nadel, 1978; Maguire et al., 1998).

Spatial navigation abilities have been shown to deteriorate as we age, often resulting in older adults avoiding unfamiliar environments which can, in turn, impact negatively on quality of life (Burns, 1999). It is widely accepted that older adults show impairments in allocentric processing and, to some extent, egocentric processing during spatial navigation tasks (Moffat, 2009; Klencklen et al., 2012; Lithfous et al., 2013; Colombo et al., 2017; Lester et al., 2017). Corresponding with declines in spatial abilities, older adults show reduced volume in the hippocampus and caudate nucleus (Raz et al., 2003; Raz and Rodrigue, 2006) which are key brain areas involved in allocentric and egocentric strategies, respectively.

Spatial navigation is also supported by the vestibular system (Brandt et al., 2005), which plays a crucial role in maintaining one’s balance and postural control (Allen et al., 2004; Angelaki and Cullen, 2008; St George and Fitzpatrick, 2011). Vestibular function also declines with age (Anson and Jeka, 2016), impacting self-motion perception and the coding of the body’s orientation and position in space. A typical finding is that older adults perform worse than their younger counterparts in a triangular completion task, i.e., returning to the starting point of a triangular path after being led along two angles of the triangle with their eyes closed (Adamo et al., 2012; Barrett et al., 2013). Even though vestibular function declines with aging, older adults rely more on vestibular information during spatial navigation, even when the visual cues are more reliable (Bates and Wolbers, 2014).

With aging, the cognitive demands of navigating through an environment may lead to resource competition between maintaining balance control and completing the navigation task, resulting in either reduced balance control (Simieli et al., 2015) or reduced performance in navigation (Lester et al., 2017), depending on task demands. This trade-off was observed in a study by Lövdén et al. (2005), in which younger and older adults walked on a treadmill while exploring a virtual museum, either with a handrail for support or without. Older adults showed a more unstable gait when navigating through the museum compared to when simply walking on the treadmill. Furthermore, age differences in spatial learning performance were more pronounced when no handrail support was supplied, whereas the performance of older adults improved in terms of both speed and accuracy with the provision of the handrail. Executive function has been shown to mediate navigational ability in aging (Taillade et al., 2013a). Navigating within a virtual environment using a joystick, balance board, or treadmill may be considered as a motor-cognitive dual-task condition, as cognitive processes are required both for motor and balance control, as well as for knowledge acquisition within the spatial environment.

Recent research in spatial navigation has benefited from the use of computer technology, with Virtual Reality (VR) in particular adopted as a means to assess spatial cognitive abilities in a wide range of groups, from healthy participants to patient studies. Relative to real-world environments, the use of virtual environments (VE) offer a number of benefits for studies of spatial cognition: VEs facilitate the study of large-scale spatial navigation within ecologically valid contexts while allowing for standardized protocols to be adopted across studies and also offer a high degree of experimental control (Diersch and Wolbers, 2019). For example, Merriman et al. (2016) embedded objects into a virtual rendering of both highly familiar and unfamiliar areas of a real university campus and reported a benefit of environment familiarity on spatial memory in older adults. Moreover, VR allows for the stimulation of multiple sensory systems (e.g., vision, audition, proprioception, vestibular system) whilst tracking and assessing the behavioral responses to the integration of different sensory cues (Dehn et al., 2018; Carr et al., 2019; Appel et al., 2020). Importantly, experimental manipulations that would be impossible in real-world scenarios of navigation can be used to understand the influence of different sensory information during specific aspects of navigation (Diersch and Wolbers, 2019).

The availability of VR has also allowed for the development of human analogs of spatial navigation tasks more typically used in rodent studies, such as the Morris Water Maze Task (Morris, 1981). The virtual Morris Water Maze Task (vWMT) has been used widely to assess spatial navigation abilities in both younger and older adults, with performance suggesting a specific decline in allocentric processing with aging (e.g., Moffat and Resnick, 2002; Antonova et al., 2009; Rodgers et al., 2012; Gazova et al., 2013; Daugherty et al., 2016). For example, Head and Isom (2010) found that older adults performed worse than younger adults on allocentric tasks embedded in a virtual maze, such as judging the temporal order of landmarks and the direction and relative distances associated with these landmarks. Wiener et al. (2013) also reported that, compared to younger adults, older adults were unable to use an allocentric spatial strategy when approaching a learned route from a novel direction or when required to repeat and retrace a learned route (Wiener et al., 2012), demonstrating allocentric but not egocentric deficits in spatial memory performance of older adults. In a novel study using dynamic VEs, Merriman et al. (2018a) reported that the presence of virtual crowds further impaired spatial memory performance in older but not younger adults. The use of VR/VE thus permits the carefully controlled study of the impact of ecologically valid everyday occurrences (i.e., crowded streets, obstacles) on older adults’ spatial memory.

Although spatial navigation abilities decline with aging, evidence is emerging that training can lead to improvements in this skill in older adults. For example, Lövdén et al. (2012) trained younger and older participants in a VE spatial navigation task combined with treadmill walking. Following 4 months of training, the authors reported reduced age-related deficits in spatial navigation in the experimental group, relative to a group that only walked on the treadmill, without spatial navigation training. Furthermore, neuroimaging suggested that the spatial memory training had a protective effect as the hippocampal volume of the intervention group of older adults remained constant between post-intervention and at 4 months follow up, while the hippocampal volume of the treadmill-only control group decreased consistent with longitudinal age-related decline (Lövdén et al., 2012). Thus, spatial navigation training appears to enhance navigation performance and protect the hippocampal structure from age-related decline. In addition, West et al. (2017) found that playing a 3D-platform video game (namely Super Mario 64©), for approximately 70 h over 6 months resulted in an increase in hippocampal gray matter volume in older adults compared to two control groups, an active, computerized music lesson group and a passive control group (West et al., 2017). Similar results were reported by Kühn et al. (2014) for a cohort of younger adults who played Super Mario 64^®^ for a period of 2 months compared to a passive control group. Importantly, the increase in hippocampal gray matter volume was associated with a shift from an egocentric strategy to an allocentric strategy, suggesting a link with hippocampal volume and performance on virtual navigation tasks. These and other studies (e.g., Hötting et al., 2013) suggest that spatial navigation training in virtual environments can confer similar benefits on spatial cognition that were previously reported for tasks involving real-world navigation (i.e., London taxi drivers, Woollett and Maguire, 2011; Brunec et al., 2019).

The current study builds on our previous findings that older adults are more adversely affected by the presence of crowds while navigating than younger adults (Merriman et al., 2018a), and other reports that attentional resources are shared between balance control and spatial navigation (Taillade et al., 2013b). Specifically, this study sought to investigate whether playing a video game that required active navigation in a 3D virtual environment of increasing complexity while avoiding obstacles would improve spatial memory performance and executive function in older adults. We developed a multicomponent intervention, named “CityQuest” that trained spatial navigation in unfamiliar, crowded environments that required the participant to use balance control to navigate through a city landscape whilst avoiding obstacles and pedestrians. To provide a better understanding of the contribution of spatial memory and the obstacle avoidance components of CityQuest, we used a component control manipulation (Boot et al., 2013) and created two control conditions, one without the spatial memory task but which involved obstacle avoidance and balance control (Obstacles-only), and the other without obstacle avoidance but which involved spatial memory and balance control (Spatial Navigation-only). While the Spatial Navigation-only condition involves dual-task training of postural stability and navigation, the CityQuest condition involves multi-task training, with an extra layer of complexity that requires additional executive functioning to avoid obstacles. We hypothesized that the “CityQuest” intervention training using a realistic, ecologically valid virtual environment would lead to improvements in spatial memory relative to spatial navigation training only or obstacle avoidance training only, due to the involvement of more cognitively demanding multi-tasking components (Van Impe et al., 2013; Moreau and Conway, 2014).

All participants were required to perform two 60-min sessions of training per week over 5 weeks, i.e., a total of 10 h of training. This time frame was based on a number of previous cognitive training studies which have suggested that 4 weeks or 10 training sessions are of sufficient duration for training gains to occur (Kelly et al., 2014; Schoene et al., 2014). Before and after the intervention, spatial memory was assessed with measures of object recognition, direction judgment (egocentric processing), and cognitive mapping abilities (allocentric processing). Furthermore, we were interested in whether spatial navigation training in general would lead to a change from a more egocentric-based navigation strategy to a more efficient allocentric spatial strategy as measured by the strategy maze assessment (see Wiener et al., 2013).

## Materials and Methods

### Participants

Participants were recruited through advertisements placed in local aging organizations and local media seeking community-dwelling adults aged 65 years or older in the Dublin area, in good general health, with no cognitive, visual, or hearing impairments, and able to maintain balance independently. A total of 70 older participants met inclusion criteria and were enrolled in the study. Fourteen participants did not complete the study: 5 withdrew due to ill health and 9 due to other commitments. Thus, the final sample included a total of 56 participants (35 female; *M* = 71.82, SD = 4.64; age range 65–84). All participants reported normal or corrected-to-normal vision and hearing, no cognitive impairment, and none reported a history of psychiatric or neurological illness. Following baseline measures (see below), participants were pseudo-randomly assigned to one of the three training intervention conditions. There were 21 participants (7 male, 14 female) assigned to the “CityQuest” training, 20 (8 male, 12 female) assigned to the “Spatial Navigation-only” training and 15 (6 male, 9 female) assigned to the “Obstacles-only” training group.

### Experimental Protocol and Design

The protocol consisted of three sets of assessments: baseline measures; pre- and post-training measures, and the training intervention itself. The overall experimental design was based on a mixed, factorial design with participant group (CityQuest, Spatial Navigation-only, or Obstacles-only) as the between group factor. Participants were enrolled on an ongoing basis and randomly allocated to the CityQuest or Spatial-navigation condition first. Participants were then recruited for the Obstacles-only condition. The experiment protocol and recruitment procedures were approved by the School of Psychology Research Ethics Committee prior to the start of the study. Accordingly, all participants provided informed, written consent prior to taking part in the experiment.

### Stimuli and Apparatus

The study took place in a dedicated testing laboratory in Trinity College Institute of Neuroscience. Two different and unique virtual environments were created for the purpose of the pre- and post-assessments: a VE for the Spatial Navigation Assessment and a VE for the Spatial Strategy Assessment task. The VEs were designed using a proprietary engine based on Ogre 3D and converted into video format. All pre- and post- training VE assessments were programmed and responses recorded using Presentation^®^ software^1^. The VE assessments were presented on a Dell Latitude E4300 laptop and viewed by the participant on a HP L1710 17″ LCD color monitor (resolution 1,024 × 768 pixels). Participants were seated approximately 57 cm in front of this monitor.

The intervention training games were presented using either a Dell Alienware Aurora 875W computer connected to a 50″ Sony Bravia LED-backlit LCD flat panel display with a refresh rate of 120 Hz, or through a Dell Optiplex 7010 computer with a refresh rate of 60 Hz connected to a standard projector directed at a white screen. This dual set-up allowed us to test two participants at the same time and participants were trained on both apparatus (i.e., each participant performed five training sessions with the LCD display and 5 with the projector display). A Wii Balance Board (WBB; Nintendo, Kyoto, Japan) was connected to each PC *via* Bluetooth. Each WBB was positioned approximately 2 m away from the display, embedded into a compliant surface mat measuring approximately 2 m × 2 m that was flush with the platform floor (for an illustration of this set-up, see Merriman et al., 2018b). For added safety, a waist-high support frame was secured around the WBB which the participant could use for support when required. The sounds from the games were presented *via* Sennheiser HD 202 headphones (we used wired and wireless versions).

#### Baseline Measures

Prior to the intervention, participants’ ability across a range of sensory and cognitive measures were measured during a “baseline” session. Measures of visual acuity and contrast sensitivity were taken using the ETDRS acuity chart and the Pelli-Robson Contrast Sensitivity Test, respectively. Hearing ability was assessed with the Hughson-Westlake Audiogram at 4 kHz. Global cognitive function was assessed using the Montreal Cognitive Assessment (MoCA) (Nasreddine et al., 2005), with performance of below a score of 23 indicative of cognitive impairment (Luis et al., 2009). Participants’ self-reported sense of direction was assessed with the Santa Barbara Sense of Direction Scale [SBSOD; *M* = 4.74, SD = 0.92; (Hegarty et al., 2002)]. This scale ranged from 1 to 7, with higher scores on this measure indicating a better sense of direction.

#### Assessment Measures (Pre- and Post-training Assessments)

We included two main tasks (described below) to assess the effectiveness and generalizability of the training intervention on aspects of spatial cognition: the Spatial Navigation Assessment task and the Spatial Strategy Assessment task. We also included a measure of executive function which was assessed using the standardized Trail Making Test (TMT) (Reitan, 1958). The performance in the TMT was evaluated by scoring the time needed for the completion of two parts, A and B. To eliminate the motor component involved in this test, both parts of the TMT were contrasted by a difference score (TMT Part B—TMT Part A) (Corrigan and Hinkeldey, 1987). The pre- and post- assessments also included measures of balance control, ratings of balance confidence and we also tested whether measures of perceptual functioning (i.e., useful field of view and motion coherence threshold assessments) were affected by the training intervention. The effect of training on measures of balance and perceptual function are reported elsewhere (O’Callaghan et al., 2018; Roudaia et al., in prep).

##### Spatial Navigation Assessment

**FIGURE 1:**
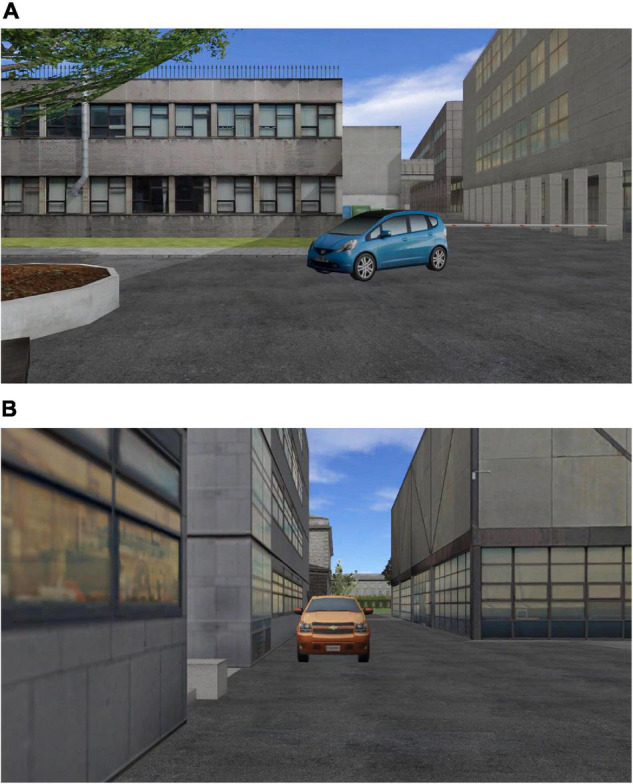
Example imagesof target objects embedded at intersections along **(A)** Route A; and **(B)** Route B of the Spatial Navigation Assessment.

The Spatial Navigation assessment was implemented in a VE that was a simulation of an area within the campus of Trinity College Dublin (Merriman et al., 2016). We created separate videos for two distinct virtual routes through this area of the VE campus and target objects were embedded at different intersections along these routes. Two separate video clips depicted a first person view of different routes taken through the VE which participants were required to learn. Each video clip was approximately 2 min in duration. Each route comprised eight intersections and at each intersection, a left turn (3 intersections), a right turn (3) or a straight ahead (2) direction was followed. Whenever the virtual camera approached an intersection within 20 m, a unique target object would appear, which participants were asked to learn (see Figures 1A,B). There were 16 target objects in all, divided into two sets and each set was allocated to one route across all participants. The presentation order of the two routes was counterbalanced across participants and across testing sessions (pre- or post-training).

Each assessment began with a learning phase, in which participants were shown a video clip of a route twice in a row and asked to remember the route and the objects encountered in the route. Directly following learning of the route, participants were tested using the following four tasks in the same sequential order, to minimize cross-over effects.

First, participants completed the Object Recognition Task, which assessed target object recall using an Old/New recognition memory design (Merriman et al., 2016). Previous research has found that the neural activity in the parahippocampal gyrus to target objects placed at decision points along a route reflects the navigational relevance of an object’s location in the learning environment. This suggests that the automatic storage of navigationally-relevant object location in the parahippocampal gyrus is part of the neural mechanism underlying successful navigation (Janzen and van Turennout, 2004). Participants were presented with either a target object or distractor object image and asked to indicate as quickly and as accurately as possible whether or not they had seen the object along the learned route by pressing one of two assigned keys (“z” and “m”) respectively, indicating a “yes” or “no” response on the keyboard. Distractors were different exemplar objects from the same category as each target object. This task consisted of 16 trials (8 targets and eight distractors) presented in random order.

Next, participants completed a Direction Judgment Task that measured egocentric spatial processing (see Head and Isom, 2010; Merriman et al., 2016). There were eight trials in this task. In each trial, participants were presented with an image of one of the eight target objects from the learned route and asked to indicate as accurately as possible whether the object was associated with a right turn, left turn or maintained a straight-ahead course by pressing one of three corresponding keys (i.e., left, up or right arrow) on a keyboard. Trial order was randomized across participants.

The third task was a “pen and paper” Target Landmark Location Task that measured allocentric processing or “cognitive mapping” (Moffat and Resnick, 2002). In this task, participants were presented with a 2D, scaled map of the learned VE campus without any target objects indicated. For each of the target objects, participants were asked to indicate its location by marking “X” on the map, without naming the target.

The fourth task was a “Target Landmark Naming” Task, in which participants were presented with another copy of the 2D, scaled map of the VE campus but this time the map was marked with “X”s which each indicated the location of a target object along the route. Participants were required to write the name of the target object at each location indicated on the map.

##### Spatial Strategy Assessment

**FIGURE 2:**
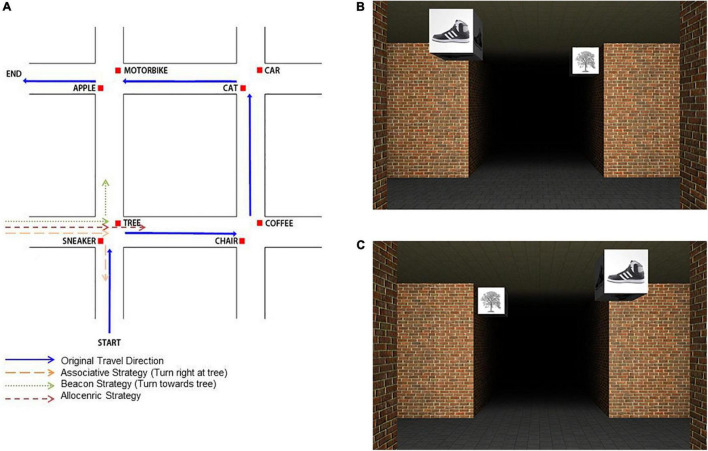
Spatial Strategy Assessment: **(A)** Schematic overview of the learning route during the spatial strategy assessment; Example image of an intersection from the maze approached from **(B)** the original learned direction (correct response: right turn); and **(C)** from a different direction to that from learning (correct response: straight ahead).

This test was designed to measure the participant’s ability to use allocentric processing for route navigation and if this strategy were more likely to be adopted following training. The test was based on a novel virtual environment which consisted of a route taken through a maze, and was adapted from Wiener et al. (2013). The maze consisted of two straight paths intersecting with two other perpendicular paths, resulting in four intersection points. The route traversed four intersections, identifiable by two unique landmarks, located on diagonally opposite corners (see Figure 2). These landmarks consisted of an image of an object presented on four sides of a cube (side length: 0.5 m), suspended 2 m above the floor. Each intersection was obscured by fog and whilst the camera approached the intersection, the object became visible from 12.5 m with a quadratic increase in light intensity.

Participants were first presented with a video clip of a first person view of a route taken through this custom-designed VE maze, populated by landmark objects, which they were required to learn. They were then tested on their spatial memory of the route.

A test trial consisted of a 6 s video clip sampled from the learned path that traversed one arm of the maze, stopping at the first intersection (see Figure 2A) and depicting the landmark objects in their original locations. These segments were presented either traveling in the same direction (same direction trials) or a different (but not reverse) direction (different direction trials) as in the learned route (see Figures 2B,C). Each clip was immediately followed by an image of white arrows pointing either left, upward, or right, prompting participants to report in which direction the learned route proceeded at this intersection by pressing the appropriate arrow key on the keyboard. There were six experimental blocks of 12 trials per block totaling 72 trials. These same and different direction trials tested different spatial processing abilities: “same direction” trials assessed egocentric strategies (associative cue or beacon strategies; Wiener et al., 2012, 2013), whereas “different direction” trials require more allocentric processing of the spatial relationships between landmarks. Spatial strategy preference was measured by calculating the percentage of responses in line with each of the three spatial strategies (associative cue, beacon and allocentric) of the “different direction” trials that distinguished between all strategies.

#### Description of the CityQuest Training Intervention

The main version of the training intervention, that is the “CityQuest” game, was custom-built and created by Testaluna© using Unity software. In the game, the participant navigates a virtual cityscape using their balance to guide the movements of their virtual avatar by shifting their weight on a Wii balance board. The participant’s task was to learn the locations of four target landmarks (e.g., pharmacy, cinema, bank, jewelers) located throughout the city during a learning phase, and then navigate to these locations using the fastest route on three subsequent game levels. At the same time, participants had to ensure that their avatar avoided obstacles in their way. The obstacles included static (e.g., puddles or bollards) or dynamic (e.g., moving balls or pedestrians) objects which were presented with semantically congruent auditory sounds (e.g., sound of rolling wheels). During game training, participants navigated from a first person perspective, however, their position was also simultaneously displayed on a smaller map of the environment presented in an aerial view from a fixed orientation in the top left-hand corner of the screen. Target landmarks were not displayed in the smaller map.

Game difficulty was adapted to each participant’s performance across sessions. The spatial navigation difficulty varied across sessions by changing the complexity of the cityscape, i.e., the number of intersections crossed in the city layout. There was a total of four complexity levels to the cityscapes corresponding to 8, 12, 16, and 22 intersections (labeled 1 to 4, respectively). Furthermore, obstacle complexity was manipulated by increasing the transparency of the obstacles, the number or density of obstacles on each street, and the speed of moving obstacles. There were eight complexity levels of obstacle avoidance in total. Performance on the game was constantly measured and points awarded (and displayed on the screen) for achievements including successful obstacle avoidance, and reaching a target landmark using the most efficient or fastest route. To ensure better learning and to sustain motivation, the difficulty and complexity in all three games was adapted to participants’ performance across different sessions (Green and Bavelier, 2008). Specifically, task difficulty was increased to the next level if the participant reached a criterion level of performance in each component (obstacle avoidance, spatial navigation). Otherwise, the participant remained at the same game level for the subsequent session. For more details of the training intervention see Merriman et al. (2018b).

##### CityQuest Training Intervention and Control Conditions

The main aim of the “CityQuest” game intervention was to locate specific target landmarks by navigating the avatar to each landmark using the most efficient route possible. Participants first learned to navigate through the virtual city by shifting their weight on a WBB to control the movements of an avatar embedded in the city. During the learning phase, participants were familiarized with the location of target landmarks within the city through active exploration. They were presented with three levels of difficulty in the game, each associated with locating the same landmarks but from different starting points within the same virtual city. During the navigation of the virtual city the participant had to also ensure that the avatar avoided obstacles by shifting their weight on the balance board.

The CityQuest game was also used as the basis for the design of two control conditions of the game, which were created to examine the effect of specific characteristics of the CityQuest game on spatial cognition and obstacle avoidance. The “Spatial Navigation-only” version of the game was identical to the CityQuest game, except that the obstacles were not included. For the “Obstacles-only” version there was no requirement to navigate to a target location. Instead, this version of the game required avoiding the same static and dynamic obstacles whilst moving through the city to collect gems located along the middle of the path within a time limit. Thus, by comparing performance in the CityQuest with performance in these control conditions, we aimed to unpick the relative contribution of spatial navigation and obstacle avoidance on the effect of the intervention on our outcome measures.

Game training performance on each session was assessed using two different measures for all training conditions, that is, the time taken at decision points and the number of intersections traversed (which was an approximation for how efficiently the cityscape was explored). There were also two different measures for the CityQuest and Spatial Navigation-only games (since the Obstacle avoidance task was time-limited) including the time taken to complete each level and navigation efficiency. Navigation efficiency was calculated by dividing the distance of the most efficient route possible to locate the target (e.g., 100 distance units) by the actual distance traveled by the participant (e.g., 130 distance units, to yield a score of 0.77) in the virtual space. Other measures of performance included obstacle avoidance efficiency (CityQuest and Obstacles-only training groups only). Obstacle avoidance efficiency was calculated as the number of obstacles successfully avoided during training divided by the total number of obstacles encountered (e.g., if 37 of the 42 objects were successfully avoided, this yielded a score of 0.88).

### Procedure

The entire study (including all baseline, assessments, and training sessions) took place over several weeks and required a site visit to a dedicated testing lab in Trinity College for the duration of the experiment. All participants first completed the baseline measures in a single session. Participants were then assigned to one of the three training groups. All participants performed the pre-assessment tests (spatial cognition, executive function, perceptual function, balance) over two different sessions. Participants were also invited to volunteer for neuroimaging (MRI) testing before and after the training sessions (for further details see Merriman, 2016; O’Callaghan et al., 2018). All participants were informed that training on the intervention required them to perform two by 60 min sessions of training per week over 5 weeks, totaling 10 training sessions. A member of the research team was present at all times during training to troubleshoot any issues raised by participants and to monitor training compliance. A minimum of a 1 day break was required between sessions to allow for sufficient levels of rest and recovery (see Montana et al., 2019). After the last training block, participants completed the post-training assessments (a repeat of the pre-training assessments).

### Data Analysis

All statistical analyses were conducted using SPSS 26 (SPSS Inc., Chicago, Illinois). The general significance level was set to *p* = 0.05 (two-tailed) unless otherwise stated. Baseline measures were analyzed using independent *t*-tests. Group differences in baseline characteristics were analyzed using a series of one-way analyses of variance (ANOVAs).

Group differences in the progress of participants’ performance through three levels of 10 training sessions on game measures were analyzed through a series of mixed ANOVAs or mixed ANCOVAs, depending on group differences in baseline characteristics, and are available under Supplementary Material. These performance measures included: (a) the amount of time taken at decision points during training; (b) the number of intersections traversed during training; (c) the time taken to complete each level during training (CityQuest and Spatial Navigation-only as time was held constant for those in Obstacles-only); (d) navigation efficiency during training (CityQuest and Spatial Navigation-only); and (e) obstacle avoidance efficiency during training (CityQuest and Obstacles-only).

The hypothesized advantage of the CityQuest game training was tested by the interaction of the factors “group” (CityQuest, Spatial Navigation-only, or Obstacles-only) and “time” (pre- and post-training) in mixed ANOVAs/ANCOVAs (depending on group differences in baseline characteristics) using performance differences across three different assessments as dependent outcome variables: accuracy to the spatial navigation assessment, accuracy to the spatial strategy assessment and time taken to complete the TMT (executive function).

For an exploration of any differences on measures of spatial navigation ability and executive function associated with improvement through training in the CityQuest and Spatial Navigation-only game conditions (i.e., progressing to at least the third difficulty level) compared either to those who did not train successfully in these conditions, or those training on the Obstacles-only condition, please see the Supplementary Material. Successful spatial navigation training was characterized by progressing to at least the 3rd difficulty level (out of a possible four difficulty levels) of the game during training.

Critical tests for distinguishing performance across groups were conducted, where appropriate, using six planned comparisons in which the effects of each training condition were compared one-on-one using paired *t*-tests at pre- and post-training, and using a Bonferroni-corrected alpha level of 0.008 (unless otherwise stated).

## Results

### Training Group Baseline Characteristics

All participants had normal or corrected to normal visual function (Sandlin et al., 2014) and hearing for their age (Müller et al., 2009; Merriman et al., 2018b). None scored below the Montreal Cognitive Assessment (MoCA) cut-off score for mild cognitive impairment (MoCA; *M* = 26.93, SD = 2.03). All participants self-reported a good sense of direction (SBSOD; *M* = 4.74, SD = 0.92).

Table 1 summarizes the results of each of the baseline measures for participants grouped by training conditions. Separate, one-way ANOVAs were conducted on each of the baseline measures with training group (3: CityQuest, Spatial Navigation-only, Obstacles-only) as the between group factor. A main effect of training group on age revealed that participants assigned to the CityQuest group were younger than those assigned to either the Spatial Navigation-only (*p* = 0.016) or Obstacles-only (*p* = 0.01) groups. There was no effect of group on MoCA score, suggesting that participants were matched in global cognitive ability. Also, there was no evidence for a group effect on participants’ rated sense of direction (SBSOD). Although visual acuity was within the normal range for all participants, a main effect of training group suggested participants in the CityQuest (*p* = 0.001) and Obstacles-only (*p* = 0.041) groups had significantly better visual acuity compared to those in the Spatial Navigation-only group. However, there was no evidence for a group difference in measures of contrast sensitivity. The groups were also matched on measures of hearing acuity.

**TABLE 1:** Mean age profile and baseline characteristics of those allocated to the CityQuest, Spatial Navigation-only and Obstacles-only training conditions (with standard deviations in parentheses).

	CityQuest (*N* = 21)	Spatial navigation-only (*N* = 20)	Obstacles-only (*N* = 15)	F ratio	*P* value
Age (years)	69.27 (2.68)	73.10 (4.59)	73.67 (5.46)	6.10	0.004*
MoCA score	27.52 (1.83)	26.85 (2.30)	26.20 (1.78)	1.94	0.154
SBSOD rating	4.63 (1.26)	4.68 (0.66)	5.00 (0.59)	0.81	0.452
Visual Acuity (LogMAR)	0.04 (0.07)	0.15 (0.12)	0.07 (0.07)	7.20	0.002*
Contrast Sensitivity (logCS)	1.95 (0.00)	1.91 (0.12)	1.95 (0.00)	2.51	0.091
Hearing Acuity (Db)	30.96 (15.36)	35.13 (16.37)	35.00 (17.32)	0.29	0.751

*significant at p < 0.05.

### Game Training Performance

Details of the results of training performance across the groups on all training measures (time taken to complete training; navigation efficiency; obstacle avoidance efficiency) can be found in Supplementary Material. In summary, although most participants improved their performance on the training game, of the 41 participants assigned to both the CityQuest and Spatial Navigation-only training conditions, 22 (10 male, 12 female; 9 CityQuest, 13 Spatial Navigation-only) successfully trained to the most difficult levels in the spatial navigation component (i.e. levels 3 and 4). Improvement in spatial navigation training was characterized by progressing to at least the third difficulty level (out of four difficulty levels) in terms of city complexity during training. In contrast, 19 (5 male, 14 female; 12 CityQuest, 7 Spatial Navigation-only) failed to improve or sufficiently progress to the required difficulty level across the 10 training sessions. Details of a series of exploratory analyses comparing performance on each of the assessment tasks between the participants whose performance improved with training and those whose performance did not improve with training can be found under Supplementary Material.

### Pre- and Post-intervention Assessment Measures

Performance on each of the pre- and post-training assessments was used to measure the effect of training, and is summarized in Table 2. As the training groups differed in age and visual acuity, these factors were initially included as covariates in the analyses of the spatial navigation assessment, spatial strategy assessment, and Trail Making Test (TMT). However, these factors did not correlate with any of the studied dependent measures (all ps = n.s.). Furthermore, there was no effect of age (all *F* ratios < 1) or visual acuity [*F* ratios < 1; landmark location task *F*(1, 51) = 1.83, *p* = 0.183, η_*p*_^2^ = 0.04; landmark naming task *F*(1, 51) = 1.72, *p* = 0.82, η_*p*_^2^ = 0.01] in any of the spatial navigation tasks, the same and different direction trials of the spatial strategy assessment, or the TMT, nor did the covariates interact with any of the dependent variables (all ps = n.s.).

**TABLE 2:** Mean performance accuracy across the spatial navigation and spatial strategy assessments, percentage strategy preference, and completion times for the trail making test at pre- and post-training across each of the training groups (with standard deviations in parentheses).

	Pre-training			Post-training		
	CityQuest	Spatial navigation-only	Obstacles-only	CityQuest	Spatial navigation-only	Obstacles-only
	*N* = 21	*N* = 19	*N* = 15	*N* = 21	*N* = 19	*N* = 15
**Spatial navigation assessment**						
Object recognition	86.01 (9.86)	85.31 (11.70)	83.75 (11.52)	91.07 (7.01)	86.25 (13.99)	92.92 (8.14)
Direction judgment	70.24 (16.52)	66.25 (21.50)	60.83 (20.52)	72.62 (18.38)	71.88 (22.53)	69.17 (15.57)
Landmark location	50.89 (22.21)	61.25 (22.73)	57.92 (22.09)	71.73 (22.93)	67.50 (26.25)	59.59 (27.74)
Landmark naming	66.07 (27.71)	60.63 (26.99)	61.67 (24.31)	66.67 (30.19)	66.88 (27.29)	64.17 (24.94)
**Spatial strategy assessment**						
Same direction	84.33 (13.56)	74.17 (21.23)	76.39 (15.40)	87.50 (10.12)	81.04 (19.84)	81.11 (13.26)
Different direction	34.92 (12.75)	31.15 (11.74)	33.75 (12.10)	36.21 (14.81)	39.37 (8.89)	36.67 (21.89)
**Strategy preference**						
Associative	47.62 (17.31)	46.25 (15.17)	45.56 (15.06)	49.21 (17.66)	45.83 (17.42)	42.78 (13.31)
Beacon	40.87 (14.17)	43.75 (12.35)	43.33 (7.18)	33.73 (17.18)	42.50 (19.85)	37.22 (14.73)
Allocentric	10.71 (15.84)	9.58 (14.38)	8.89 (12.39)	15.87 (19.53)	10.42 (14.27)	18.89 (19.79)
**Trail making test**						
Completion time (B-A)	54.93 (20.72)	60.60 (43.95)	57.53 (26.09)	35.31 (12.07)	48.02 (37.49)	49.93 (23.92)

#### Spatial Navigation Assessment

See Table 2 for mean performance accuracy across training groups on the spatial navigation assessments. We hypothesized that those in the CityQuest and Obstacles-only group would improve across pre- and post-training assessments on the object recognition task within the Spatial Navigation assessment, as training involved recognizing obstacles to avoid under increasing levels of difficulty which was common to both groups. For the remaining tasks in the spatial navigation assessment, we expected that those in the CityQuest and Spatial Navigation-only groups would perform better than those in the Obstacles-only group across pre- and post-training assessment of spatial navigation.

A series of mixed ANOVAs with group (3: CityQuest, Spatial Navigation-only, Obstacles-only) as the between group factor and time (2: pre-, post-training) as the within group factor were conducted on performance accuracy to the object recognition task, direction judgment task, landmark location task, and landmark naming task in the spatial navigation assessment. A series of one-way ANOVAs confirmed no differences at pre-training assessment among the training groups on the spatial navigation assessment, spatial strategy assessment, or TMT [all ps > 0.15].

An ANOVA of performance on the object recognition task showed no main effect training group [*F*(2, 53) < 1]. There was a main effect of time [*F*(1, 53) = 7.49, *p* = 0.008, η_*p*_^2^ = 0.12], with better performance post- (*M* = 89.84, SD = 10.5) than pre- training assessment (*M* = 85.16, SD = 10.83, *p* = 0.014). There was no evidence for an interaction between training group and time [*F*(2, 53) = 1.57, *p* = 0.22, η_*p*_^2^ = 0.06].

An analysis of performance accuracy on the direction judgment task, revealed no effect of training group [*F*(2, 53) < 1], no effect of time [*F*(1, 53) = 3.07, *p* = 0.086, η_*p*_^2^ = 0.06], and no interaction between training group and time [*F*(2, 53) < 1].

An ANOVA of performance on the landmark location task revealed no effect of training group [*F*(2, 53) < 1]. There was a main effect of time [*F*(1, 53) = 9.87, *p* = 0.003, η_*p*_^2^ = 0.16], with performance on this assessment improving from pre- (*M* = 56.47, SD = 22.42) to post- training (*M* = 66.96, SD = 25.47, *p* = 0.002), and a significant interaction between training group and time [*F*(1 = 2, 53) = 3.7, *p* = 0.031, η_*p*_^2^ = 0.12]. The CityQuest group improved in performance on this task from pre- to post-training training (*p* = 0.001), whereas there was no such improvement found for the Spatial Navigation-only (*p* = 0.16) and Obstacles-only (*p* = 0.8) groups.

Finally, an analysis of performance on the landmark naming task revealed no effect of training group, no effect of time and no interaction between these factors [all *F* ratios < 1].

#### Spatial Strategy Assessment

We hypothesized that those who received spatial navigation training would perform better than those in the Obstacles-only group across pre- and post-training assessments on the same direction and different direction trials. In particular we expected that those in the Spatial Navigation-only group would show greater improvement than those assigned to the other two training groups on the different direction trials as their training took place in an empty city, similar to the empty corridors utilized in this maze assessment (see Figures 2B,C). We were also interested in whether strategy preference would change across groups as a result of training. For the spatial strategy assessment, a series of mixed ANOVAs were carried out on performance accuracy to the same and different direction trials and in the spatial strategy preference analysis. See Table 2 for mean performance accuracy across training groups on this spatial strategy assessment measures.

The ANOVA on performance to the “same direction” trials showed no effect training group [*F*(2, 53) = 2.18, *p* = 0.12, η_*p*_^2^ = 0.08]. There was a main effect of time [*F*(1, 53) = 4.56, *p* = 0.037, η_*p*_^2^ = 0.08], with performance improving from pre- (*M* = 78.57, SD = 17.43) to post-training (*M* = 83.48, SD = 15.1, *p* = 0.033). There was no interaction between the training group and time [*F*(2, 53) < 1].

An analysis of performance to the “different direction” trials suggested that it was generally poor: performance across the three training conditions was not significantly better than chance (33%) at either pre-training or post-training [all *t* values < 1], with the exception of the performance of the Spatial Navigation-only group at post-training [*t*(19) = 3.21, *p* = 0.005]. An analysis of performance to the “different direction” trials revealed no effect training group [*F*(2, 53) < 1]. However, there was a main effect of time [*F*(1, 53) = 4.26, *p* = 0.044, η_*p*_^2^ = 0.07], with performance significantly improving from pre- (*M* = 33.26, SD = 12.11) to post-training (*M* = 37.46, SD = 15.2, *p* = 0.04) across all groups. There was no interaction between training group and time [*F*(2, 53) = 1.19, *p* = 0.31, η_*p*_^2^ = 0.04].

To assess participants’ preferred strategy for navigation, for each participant we calculated the percentage of their responses which were consistent with each of the three navigation strategies (i.e., use of an associative cue, beacon, or allocentric strategy) in the “different direction” trials that distinguished between all strategies (see example in Figures 2B,C), at pre- and post-training. We conducted a mixed ANOVA with training group (3) as the between group factor, and time (2: pre-, post-training) and strategy type (3: associative cue, beacon, allocentric) as the within group factors. There was no effect of group or time [all *F* ratios < 1] but a main effect of strategy was found [*F*(2, 52) = 62.06, *p* < 0.001, η_*p*_^2^ = 0.71]. This main effect indicated a greater preference for the associative cue (*M* = 46.43, SD = 11.82) and beacon strategies (*M* = 40.18, SD = 11.5) compared to the allocentric strategy (*M* = 12.28, SD = 13.49, *p* < 0.001) for the participants. There were no interactions between group and time [*F*(2, 52) < 1] nor between strategy and time [*F*(2, 52) = 3.06, *p* = 0.055, η_*p*_^2^ = 0.11]. There were no other significant interactions found (all *F* ratios < 1).

#### Training Effect on Executive Function

We hypothesized that executive function performance of those allocated to the CityQuest condition would improve across pre- and post-training as this condition involved a higher level of multitasking than the Spatial Navigation-only or Obstacles-only conditions. Performance across groups on the TMT was analyzed pre- and post-training with a mixed ANOVA using the difference score (TMT B—TMT A) described above. The mixed ANOVA showed no effect of training group [*F*(2, 51) < 1]. There was a main effect of time [*F*(1, 51) = 11.54, *p* = 0.001, η_*p*_^2^ = 0.18], with performance improving from pre- (*M* = 57.65, SD = 31.56) to post-training (*M* = 43.84, SD = 26.93, *p* = 0.001). There was no interaction between training group and time [*F*(2, 51) < 1]. However, planned comparisons revealed that the CityQuest trained group performed the TMT more quickly following training (post-assessment stage) than before training (*p* = 0.005) compared to either the Spatial Navigation-only (*p* = 0.042) or Obstacles-only (*p* = 0.39) groups.

## Discussion

This study was designed to investigate whether a spatial navigation and obstacle avoidance intervention, coupled with a balance control component using the Wii balance board, improved spatial memory performance and executive function in older adults. To that end, participants embarked on a training intervention over several weeks and their performance was compared to participants enrolled in one of two control conditions involving training on spatial navigation only or obstacle avoidance only. Our findings indicated that all three training conditions resulted in improvements for older adults in general on different but not all outcome measures, namely the object recognition task, the landmark location task, different direction trials of the spatial strategy assessment, and executive function.

### Assessment of Spatial Navigation Following Training

The CityQuest intervention condition contained multiple components (i.e., locating target landmarks while avoiding obstacles and maintaining balance), a training approach which has been shown to result in the most effective cognitive enhancement of older adults (Basak et al., 2008; Anguera et al., 2013). We expected older adults allocated to the CityQuest group and the Spatial Navigation-only group to improve on all measures of the spatial navigation task, but not those in the Obstacles-only group, as their training did not have a spatial learning component. However, we found no improvement on the direction judgment or landmark naming tasks in the performance of any of the groups following training. One reason that may account for this lack of improvement is that performance was already quite good on both of these tasks for all training groups at the pre-training stage (66 and 64%, respectively per assessment) and it is possible that older adults had reached ceiling effects in terms of their performance (Whitlock et al., 2012).

We hypothesized that following training, both the CityQuest and Obstacles-only group would improve on the object recognition task relative to the Spatial Navigation-only group since object avoidance was common to their interventions. While the performance of the Obstacles-only group improved significantly following training, only a modest but non-significant performance improvement was found in the CityQuest group. During training, participants in the Obstacles-only group had greater obstacle avoidance efficiency (see Supplementary Material) which may explain their relatively better performance on the post-training, object recognition assessment. Moreover, this group was trained to focus specifically on the objects they encountered whilst the layout of the VE was task irrelevant. In contrast, those trained in spatial navigation focused on remembering the routes to the various landmarks during training and the city layout, without a requirement to remember specific obstacles. There may, therefore, have been a difference in the allocation of cognitive resources across groups: the Obstacle-avoidance group may have focused more on objects, whereas the spatial navigation groups focused more on the route. Although the spatial navigation groups did not improve their object recognition performance, the results from the current study indicate that it is nevertheless possible to improve performance in older adults, such as object recognition, using training that is targeted at a specific cognitive domain.

Age-related change in spatial abilities may be due in part to declines in general cognitive function, such as attention and working memory, speed of processing, executive function etc. (Sanders et al., 2008; Lester et al., 2017). However, not all spatial abilities show the same pattern of age-related decline, suggesting that global cognitive factors do not fully characterize specific spatial memory deficits as we get older (Lester et al., 2017; Yamamoto et al., 2019). Relatively preserved egocentric processing in older adults has been widely reported (Wiener et al., 2012; Gazova et al., 2013; Montefinese et al., 2015; Colombo et al., 2017; Fricke and Bock, 2018), particularly when compared to allocentric processing (Merriman et al., 2016, 2018a; Ruggiero et al., 2016; Caffò et al., 2020). Reliance on egocentric spatial strategies may represent a less cognitively demanding approach to achieve successful navigation and may constitute a strategic way to compensate for an age-related decline in both allocentric processing and general cognition, particularly of attentional and executive functioning (Colombo et al., 2017).

The CityQuest condition was designed to target several cognitive abilities (i.e., spatial navigation, obstacle avoidance, balance control), therefore we predicted a broader transfer of training benefits from this condition than the more focused Spatial Navigation-only or Obstacles-only conditions (Lustig et al., 2009; Moreau and Conway, 2013). As predicted, those assigned to the CityQuest group showed significant improvement on the landmark location task compared to those in the Spatial Navigation-only or Obstacles-only groups, although this group difference did not generalize to the landmark naming task. However, those who showed improvement in spatial navigation training performed significantly better on both the landmark location task and landmark naming task compared to those who did not improve in spatial navigation training (see Supplementary Material). The ability to recall the location of target landmarks presented on a 2D survey view map is considered a measure of “cognitive mapping” ability (Moffat and Resnick, 2002). During game training, all training groups navigated from first person perspective, however, their position was also simultaneously displayed on a smaller map of the environment presented in an aerial view from a fixed orientation. This 2D map may have provided sufficient visual cues to complete the task by associating egocentric directional information with a location. For example, the 2D map contained an outline of the buildings in the area which may have provided an adequate context to elicit an association between a particular target object with a given location. Therefore, it is unclear whether participants may have referred to the aerial map to recall target locations from an egocentric perspective or relied on their own allocentric cognitive map for the landmark location and naming tasks. Indeed, impairments in spatial learning may be emphasized in tasks requiring the processing of multiple orientations of an environment (i.e., survey, first person) during spatial memory formation (Yamamoto et al., 2019). Performance in the spatial strategy assessment task may therefore be more insightful regarding the type of spatial strategy adopted that led to improvement on the landmark location and naming tasks.

### Spatial Strategy Assessment

Wiener et al. (2013) previously reported that older adults rely more on an egocentric strategy when an allocentric strategy is required for successful navigation. The results of the current study support their finding, with egocentric strategies more likely to be adopted, such as associative cue or beacon following training, as opposed to an allocentric strategy. For the “same direction” trials of the strategy assessment, navigation can be most efficiently solved using an egocentric spatial strategy. Performance of older adults in general was quite good on the same direction trials (78%), indicating that they could successfully judge the direction to be taken based on their recall of the original route during the learning phase. As with the direction judgment task in the spatial navigation assessment (also a measure of egocentric processing), it is possible that older adults’ performance was at ceiling prior to the training thus there was effectively little room for improvement on this task following training.

However, some evidence suggests that older adults tend to rely on an egocentric strategy during many spatial navigation tasks, even if an allocentric spatial strategy would be more efficient (Head and Isom, 2010; Rodgers et al., 2012; Wiener et al., 2013). Performance on the different direction trials in the current study was quite poor for older adults in general, remaining at chance level prior to training, with only those in the Spatial Navigation-only training group reaching levels above chance at the post-training assessment. This result suggests that even those older adults who performed well on route learning on the same direction trials were unable to form a cognitive map and utilize an allocentric strategy to solve the task when the route was approached from an unfamiliar direction. Although the performance in the different direction trials of some older adults (particularly those allocated to the Spatial Navigation-only condition and those who showed improvement in spatial navigation training, see Supplementary Material) improved across assessments, there was no obvious change in their strategy preference from an associative cue one to an allocentric strategy as a result of spatial navigation training. Therefore it is possible that those whose performance improved on different direction trials did so using an egocentric spatial strategy rather than a switch to an allocentric strategy.

### Executive Function

Although executive function and working memory are different cognitive domains, both play an important role in successful spatial navigation, e.g., selecting the correct spatial strategy, switching to alternative strategies when appropriate, maintaining navigational goals, computing directions and distances to goals, translation of spatial representations (Wolbers and Hegarty, 2010). Similarly Weisberg and Newcombe (2016) provided evidence that integrators and non-integrators of spatial information have better verbal and spatial working memory performance than imprecise navigators. Therefore age-related spatial memory deficits for large-scale environments could partially be the consequence of reduced executive functioning and working memory function (Colombo et al., 2017). Executive function has also been shown to mediate navigational ability in aging (Taillade et al., 2013b). As the CityQuest condition is a complex, multitask training condition, we anticipated that training on this condition would significantly improve performance on executive function typically measured using the Trail Making Test (TMT). We found that while all participants slightly improved on the TMT following training, the performance of the CityQuest group significantly improved from pre- to post-assessment.

### Vestibular Contributions

The CityQuest training intervention also included a balance control component, where participants had to shift their weight on the WBB to control the location of the virtual character to locate target landmarks and avoid obstacles within the virtual environment. The inclusion of a postural control element while training spatial navigation adds to the ecological validity of the intervention as real-life navigation is a complex motor-cognitive dual-task, where attentional resources need to be allocated to both motor control and spatial knowledge acquisition at the same time. To examine the effect of training on cortical areas involved in motor control and the vestibular system, a subset of participants were scanned with MRI following the baseline assessment and at completion of the study. These neuroimaging findings along with additional measures of balance control and balance confidence were reported by O’Callaghan et al. (2018). While no significant differences were found across the balance control and confidence measure, we found that successful completion of the intervention training was associated with an increase in gray matter volume in the precentral gyrus (an area associated with motor control) for all participants. The precentral gyrus is subject to age-related atrophy (Good et al., 2001, 2002; Lemaître et al., 2005), however, our findings demonstrated that spatial navigation training which incorporates body movements associated with balance control can attenuate the aging effect on this cortical region important for vestibular functioning.

### Implications for Future Research

While this study demonstrated transfer of training effects of those allocated to the CityQuest and Spatial Navigation-only conditions to some measures of spatial memory and to a measure of executive function, we did not find that training benefited performance across all spatial tasks. One possible reason for this may include the duration of training. While a number of reviews of cognitive training studies have suggested that 4 weeks or 10 training sessions may be sufficient for training gains to occur (see Kelly et al., 2014; Schoene et al., 2014), other studies have found that longer interventions show greater training benefits. For example Basak et al. (2008) found that while 23.5 h of training was sufficient to observe beneficial effects of cognitive training in older adults, an assessment carried out mid-way through training (i.e., following 12 h of training) revealed 12 h of training was insufficient for any training benefit to be found. Similarly Stern et al. (2011) found that relative to the ten sessions used in training younger adults on an executive function game named “Space Fortress,” it was necessary to increase the duration of the intervention to three times longer when training older adults. A review of the use of virtual environments to train spatial abilities in stroke patients found that 8–15 training sessions of between 40–45 min duration were sufficient to show training benefits (Montana et al., 2019). However, delivering a spatial navigation intervention over a shorter time period but more intensely (e.g., 10 sessions over a 2 week instead of 5 week period) may also lead to improvements in spatial abilities (McLaren-Gradinaru et al., 2020).

Future research should aim to determine whether increasing the hours of training and/or the intensity of training would induce transfer to other tasks or lead the unsuccessfully trained to improve their navigation efficiency. Furthermore the CityQuest condition, which trained spatial navigation, obstacle avoidance, and balance control simultaneously could be considered “full emphasis training,” where all components of the training are given equal priority (Gopher et al., 1989). However, some studies have shown that the use of variable priority training, where participants are instructed to play the entire game at all times, but to shift their emphasis to different components of the game at different times during training led to greater transfer of a training benefit to untrained tasks (Gopher et al., 1989; Kramer et al., 1995; Silsupadol et al., 2009; Boot et al., 2010; Stern et al., 2011). Future iterations of the CityQuest game should apply this approach in order to investigate whether variable priority training might result in greater transfer to the outcome measures assessed.

## Conclusion

In sum, our findings add to the literature on cognitive training interventions in that an intervention involving a video game incorporating spatial navigation and obstacle avoidance training with a balance control component in a virtual environment was successful in improving egocentric spatial processing and executive function in older adults. Training in spatial navigation does not facilitate a switch from egocentric to allocentric spatial strategies in older adults, but may lead to more efficient use of egocentric spatial strategies. Furthermore, spatial navigation training within an ecologically valid virtual environment complete with obstacles to avoid can result in more performance gains than training in an empty unpopulated virtual environment.

## Data Availability Statement

The raw data supporting the conclusions of this article will be made available by the authors, without undue reservation.

## Ethics Statement

The studies involving human participants were reviewed and approved by School of Psychology Research Ethics Committee, Trinity College Dublin, Ireland. The patients/participants provided their written informed consent to participate in this study.

## Author Contributions

NM and ER designed the experiment and collected the data. JO, CO’S, MR, and IO developed the virtual environments used for assessment and training. NM wrote the manuscript, performed the data and statistical analysis with assistance on approach and interpretation from ER and FN. FN critically evaluated the manuscript. All authors contributed to the article and approved the submitted version.

## Conflict of Interest

IO was employed by company Testaluna. The remaining authors declare that the research was conducted in the absence of any commercial or financial relationships that could be construed as a potential conflict of interest.

## Publisher’s Note

All claims expressed in this article are solely those of the authors and do not necessarily represent those of their affiliated organizations, or those of the publisher, the editors and the reviewers. Any product that may be evaluated in this article, or claim that may be made by its manufacturer, is not guaranteed or endorsed by the publisher.

## Footnotes

1
http://www.neurobs.com
