# Supplementary material for: “CityQuest,” A Custom-Designed Serious Game, Enhances Spatial Memory Performance in Older Adults
Source: Front Aging Neurosci. 2022 Mar 8;14:806418. doi: 10.3389/fnagi.2022.806418 (PMC8959141; doi:10.3389/fnagi.2022.806418)
Supplement: Supplementary file 1 [file Data_Sheet_1.docx]

‘CityQuest’, a custom-designed serious game, enhances spatial memory performance in older adults

Supplementary Material

**Supplemental Table 1:** Game performance accuracy across training groups on game performance measures: mean stop time at decision points, mean number of intersections traversed, time to completion, navigation efficiency, and obstacle avoidance efficiency.

|  |  |  | **Session 1** | | | **Session 2** | | | **Session 3** | | |
| --- | --- | --- | --- | --- | --- | --- | --- | --- | --- | --- | --- |
|  |  |  | **Level 1** | **Level 2** | **Level 3** | **Level 1** | **Level 2** | **Level 3** | **Level 1** | **Level 2** | **Level 3** |
| **Stop time at decision points** | **CityQuest** | **Mean** | 3.13 | 2.86 | 2.83 | 2.73 | 2.69 | 2.84 | 2.49 | 2.38 | 2.27 |
|  |  | **SD** | 0.77 | 0.71 | 0.79 | 0.71 | 1.02 | 1.19 | 1.00 | 0.92 | 0.68 |
|  | **Spatial Navigation-only** | **Mean** | 3.61 | 2.99 | 3.22 | 2.76 | 2.57 | 2.68 | 2.34 | 2.40 | 2.10 |
|  |  | **SD** | 1.84 | 1.10 | 1.68 | 1.53 | 1.17 | 1.28 | 1.08 | 1.16 | 0.75 |
|  | **Obstacles-only** | **Mean** | 3.87 | 2.28 | 2.23 | 1.84 | 1.83 | 1.64 | 1.53 | 1.49 | 1.42 |
|  |  | **SD** | 2.44 | 1.11 | 0.93 | 0.69 | 0.67 | 0.65 | 0.56 | 0.51 | 0.51 |
|  |  |  |  |  |  |  |  |  |  |  |  |
| **No of intersections traversed** | **CityQuest** | **Mean** | 39.67 | 41.45 | 31.24 | 33.57 | 30.31 | 31.19 | 31.21 | 30.02 | 23.86 |
|  |  | **SD** | 17.67 | 20.04 | 14.03 | 15.94 | 15.06 | 13.32 | 13.29 | 19.74 | 11.66 |
|  | **Spatial Navigation-only** | **Mean** | 34.60 | 31.68 | 28.10 | 36.20 | 30.65 | 28.25 | 32.83 | 30.10 | 26.00 |
|  |  | **SD** | 16.85 | 18.44 | 15.51 | 19.90 | 18.72 | 14.07 | 15.05 | 11.67 | 15.22 |
|  | **Obstacles-only** | **Mean** | 39.40 | 40.57 | 33.23 | 49.20 | 43.47 | 38.93 | 51.20 | 46.00 | 39.57 |
|  |  | **SD** | 7.83 | 9.81 | 9.41 | 7.04 | 8.40 | 5.74 | 10.42 | 5.29 | 6.67 |
|  |  |  |  |  |  |  |  |  |  |  |  |
| **Time to level completion** | **CityQuest** | **Mean** | 161.55 | 162.14 | 129.88 | 120.27 | 110.15 | 112.29 | 106.99 | 103.45 | 84.08 |
|  |  | **SD** | 65.57 | 63.33 | 52.97 | 54.23 | 49.41 | 44.04 | 39.70 | 60.15 | 36.83 |
|  | **Spatial Navigation-only** | **Mean** | 147.56 | 126.51 | 112.82 | 143.23 | 112.92 | 107.30 | 111.42 | 105.72 | 88.01 |
|  |  | **SD** | 57.31 | 65.03 | 51.18 | 65.69 | 52.01 | 37.32 | 55.60 | 45.61 | 46.13 |
|  |  |  |  |  |  |  |  |  |  |  |  |
| **Navigation efficiency** | **CityQuest** | **Mean** | 0.45 | 0.39 | 0.43 | 0.53 | 0.54 | 0.53 | 0.52 | 0.57 | 0.63 |
|  |  | **SD** | 0.12 | 0.19 | 0.16 | 0.16 | 0.18 | 0.14 | 0.14 | 0.16 | 0.16 |
|  | **Spatial Navigation-only** | **Mean** | 0.50 | 0.54 | 0.56 | 0.42 | 0.52 | 0.50 | 0.51 | 0.52 | 0.56 |
|  |  | **SD** | 0.17 | 0.20 | 0.15 | 0.19 | 0.22 | 0.21 | 0.18 | 0.15 | 0.23 |
|  |  |  |  |  |  |  |  |  |  |  |  |
| **Obstacle avoidance efficiency** | **CityQuest** | **Mean** | 0.82 | 0.73 | 0.70 | 0.83 | 0.78 | 0.73 | 0.79 | 0.82 | 0.82 |
|  |  | **SD** | 0.09 | 0.10 | 0.14 | 0.11 | 0.10 | 0.13 | 0.12 | 0.10 | 0.08 |
|  | **Obstacles-only** | **Mean** | 0.78 | 0.83 | 0.71 | 0.84 | 0.78 | 0.77 | 0.80 | 0.80 | 0.78 |
|  |  | **SD** | 0.09 | 0.06 | 0.11 | 0.09 | 0.07 | 0.10 | 0.07 | 0.08 | 0.10 |

| **Session 4** | | | **Session 5** | | | **Session 6** | | | **Session 7** | | |
| --- | --- | --- | --- | --- | --- | --- | --- | --- | --- | --- | --- |
| **Level 1** | **Level 2** | **Level 3** | **Level 1** | **Level 2** | **Level 3** | **Level 1** | **Level 2** | **Level 3** | **Level 1** | **Level 2** | **Level 3** |
| 2.08 | 2.09 | 1.99 | 2.17 | 2.04 | 1.90 | 1.96 | 1.92 | 1.88 | 2.04 | 1.96 | 1.88 |
| 0.61 | 0.71 | 0.61 | 0.68 | 0.63 | 0.62 | 0.55 | 0.77 | 0.76 | 0.54 | 0.72 | 0.61 |
| 2.20 | 2.16 | 1.92 | 2.01 | 1.87 | 1.72 | 2.09 | 2.06 | 1.81 | 1.85 | 1.75 | 1.75 |
| 1.16 | 0.83 | 0.73 | 0.81 | 0.76 | 0.61 | 0.94 | 0.96 | 0.89 | 0.73 | 0.73 | 0.91 |
| 1.42 | 1.43 | 1.44 | 1.38 | 1.32 | 1.40 | 1.40 | 1.25 | 1.24 | 1.13 | 1.12 | 1.09 |
| 0.52 | 0.63 | 0.60 | 0.69 | 0.67 | 0.83 | 0.75 | 0.60 | 0.52 | 0.40 | 0.39 | 0.40 |
|  |  |  |  |  |  |  |  |  |  |  |  |
| 30.81 | 29.99 | 29.52 | 35.19 | 24.88 | 29.64 | 35.69 | 32.38 | 33.55 | 33.21 | 28.17 | 28.50 |
| 19.06 | 14.56 | 19.23 | 18.08 | 19.05 | 14.28 | 25.46 | 18.84 | 24.62 | 14.40 | 12.46 | 19.04 |
| 33.79 | 23.86 | 22.32 | 31.10 | 26.98 | 26.83 | 32.33 | 31.10 | 26.28 | 39.08 | 28.28 | 28.45 |
| 25.03 | 8.84 | 15.20 | 14.22 | 13.04 | 15.13 | 15.47 | 15.98 | 14.40 | 17.92 | 13.12 | 14.79 |
| 46.93 | 46.17 | 38.60 | 48.20 | 43.73 | 39.09 | 46.93 | 44.97 | 39.30 | 46.20 | 43.53 | 39.87 |
| 7.62 | 6.69 | 3.88 | 10.82 | 4.70 | 5.18 | 8.03 | 11.51 | 7.75 | 8.82 | 8.08 | 8.77 |
|  |  |  |  |  |  |  |  |  |  |  |  |
| 106.35 | 99.12 | 97.64 | 121.83 | 94.89 | 102.26 | 118.08 | 104.38 | 116.46 | 106.08 | 90.36 | 88.99 |
| 61.54 | 41.78 | 51.61 | 71.78 | 82.89 | 44.99 | 82.93 | 67.99 | 102.50 | 48.61 | 38.60 | 63.79 |
| 112.87 | 79.44 | 70.53 | 95.41 | 82.74 | 78.73 | 103.01 | 98.13 | 80.86 | 112.28 | 80.04 | 77.93 |
| 98.26 | 31.73 | 36.21 | 36.43 | 36.68 | 34.45 | 45.44 | 53.62 | 41.51 | 62.02 | 34.04 | 28.04 |
|  |  |  |  |  |  |  |  |  |  |  |  |
| 0.59 | 0.56 | 0.60 | 0.60 | 0.64 | 0.60 | 0.59 | 0.61 | 0.63 | 0.56 | 0.60 | 0.63 |
| 0.20 | 0.22 | 0.23 | 0.15 | 0.20 | 0.20 | 0.20 | 0.19 | 0.21 | 0.16 | 0.18 | 0.20 |
| 0.59 | 0.66 | 0.70 | 0.58 | 0.65 | 0.64 | 0.62 | 0.64 | 0.68 | 0.56 | 0.64 | 0.65 |
| 0.23 | 0.17 | 0.22 | 0.23 | 0.21 | 0.22 | 0.16 | 0.20 | 0.18 | 0.22 | 0.21 | 0.17 |
|  |  |  |  |  |  |  |  |  |  |  |  |
| 0.83 | 0.78 | 0.76 | 0.84 | 0.79 | 0.80 | 0.83 | 0.80 | 0.77 | 0.83 | 0.78 | 0.78 |
| 0.10 | 0.12 | 0.10 | 0.07 | 0.20 | 0.11 | 0.08 | 0.12 | 0.08 | 0.10 | 0.10 | 0.10 |
| 0.85 | 0.85 | 0.81 | 0.80 | 0.85 | 0.80 | 0.85 | 0.85 | 0.84 | 0.84 | 0.87 | 0.85 |
| 0.06 | 0.07 | 0.06 | 0.06 | 0.08 | 0.08 | 0.04 | 0.05 | 0.06 | 0.05 | 0.05 | 0.04 |

| **Session 8** | | | **Session 9** | | | **Session 10** | | |
| --- | --- | --- | --- | --- | --- | --- | --- | --- |
| **Level 1** | **Level 2** | **Level 3** | **Level 1** | **Level 2** | **Level 3** | **Level 1** | **Level 2** | **Level 3** |
| 2.13 | 1.94 | 1.98 | 1.88 | 1.77 | 1.81 | 1.92 | 1.78 | 1.93 |
| 0.65 | 0.77 | 0.70 | 0.66 | 0.44 | 0.62 | 0.58 | 0.52 | 0.64 |
| 1.69 | 1.65 | 1.60 | 1.74 | 1.67 | 1.51 | 1.64 | 1.66 | 1.51 |
| 0.81 | 0.57 | 0.64 | 0.61 | 0.59 | 0.51 | 0.57 | 0.66 | 0.58 |
| 1.14 | 1.09 | 1.13 | 1.08 | 0.94 | 1.01 | 1.04 | 0.95 | 1.04 |
| 0.47 | 0.50 | 0.54 | 0.51 | 0.41 | 0.42 | 0.55 | 0.44 | 0.54 |
|  |  |  |  |  |  |  |  |  |
| 36.57 | 30.10 | 31.83 | 35.98 | 44.62 | 30.26 | 36.79 | 35.29 | 35.55 |
| 14.35 | 17.06 | 21.20 | 19.15 | 30.77 | 16.66 | 18.61 | 24.16 | 18.65 |
| 44.18 | 31.11 | 26.87 | 38.28 | 38.10 | 31.80 | 50.10 | 30.63 | 33.97 |
| 22.09 | 21.80 | 16.09 | 20.76 | 22.62 | 12.87 | 43.45 | 14.14 | 18.10 |
| 50.90 | 43.63 | 38.43 | 50.23 | 44.90 | 38.73 | 49.40 | 46.23 | 38.90 |
| 11.57 | 7.15 | 9.37 | 9.39 | 5.52 | 5.23 | 6.17 | 6.49 | 5.85 |
|  |  |  |  |  |  |  |  |  |
| 113.84 | 95.75 | 106.24 | 97.61 | 119.00 | 89.49 | 95.61 | 95.43 | 91.50 |
| 41.86 | 60.82 | 73.51 | 51.49 | 70.49 | 41.10 | 36.47 | 50.09 | 31.45 |
| 107.69 | 83.30 | 74.37 | 88.50 | 90.87 | 75.00 | 110.26 | 79.13 | 85.74 |
| 44.57 | 53.24 | 45.24 | 40.01 | 53.40 | 31.02 | 99.22 | 46.90 | 55.68 |
|  |  |  |  |  |  |  |  |  |
| 0.55 | 0.61 | 0.63 | 0.64 | 0.60 | 0.65 | 0.61 | 0.62 | 0.62 |
| 0.17 | 0.19 | 0.19 | 0.16 | 0.17 | 0.16 | 0.19 | 0.19 | 0.19 |
| 0.57 | 0.68 | 0.69 | 0.61 | 0.64 | 0.64 | 0.62 | 0.68 | 0.69 |
| 0.21 | 0.24 | 0.24 | 0.21 | 0.24 | 0.25 | 0.21 | 0.20 | 0.23 |
|  |  |  |  |  |  |  |  |  |
| 0.83 | 0.81 | 0.78 | 0.86 | 0.82 | 0.77 | 0.86 | 0.84 | 0.80 |
| 0.07 | 0.11 | 0.09 | 0.10 | 0.07 | 0.12 | 0.07 | 0.08 | 0.08 |
| 0.82 | 0.89 | 0.84 | 0.85 | 0.89 | 0.85 | 0.84 | 0.91 | 0.84 |
| 0.05 | 0.03 | 0.05 | 0.05 | 0.03 | 0.04 | 0.06 | 0.06 | 0.04 |

**Supplemental Table 2:** Game performance accuracy across Improvement groups on game performance measures: mean stop time at decision points, mean number of intersections traversed, time to completion, navigation efficiency, and obstacle avoidance efficiency.

|  |  |  | **Session 1** | | | **Session 2** | | | **Session 3** | | |
| --- | --- | --- | --- | --- | --- | --- | --- | --- | --- | --- | --- |
|  |  |  | **Level 1** | **Level 2** | **Level 3** | **Level 1** | **Level 2** | **Level 3** | **Level 1** | **Level 2** | **Level 3** |
| **Stop time at decision points** | **Improved** | **Mean** | 3.51 | 2.78 | 2.97 | 2.83 | 2.65 | 2.85 | 2.59 | 2.40 | 2.01 |
|  |  | **SD** | 1.71 | 0.98 | 1.56 | 1.40 | 1.20 | 1.37 | 1.28 | 1.16 | 0.68 |
|  | **Not improved** | **Mean** | 3.20 | 3.09 | 3.08 | 2.65 | 2.61 | 2.66 | 2.21 | 2.38 | 2.40 |
|  |  | **SD** | 0.94 | 0.82 | 0.96 | 0.85 | 0.98 | 1.06 | 0.60 | 0.89 | 0.70 |
|  |  |  |  |  |  |  |  |  |  |  |  |
| **No of intersections traversed** | **Improved** | **Mean** | 32.41 | 34.50 | 29.59 | 26.91 | 25.91 | 22.18 | 26.91 | 27.00 | 18.25 |
|  |  | **SD** | 15.29 | 18.93 | 15.31 | 12.18 | 15.60 | 8.36 | 12.51 | 11.84 | 10.46 |
|  | **Not improved** | **Mean** | 42.74 | 39.21 | 29.84 | 44.05 | 35.76 | 38.53 | 37.89 | 33.61 | 32.61 |
|  |  | **SD** | 18.11 | 20.71 | 14.31 | 19.10 | 16.83 | 13.35 | 13.64 | 19.70 | 12.41 |
|  |  |  |  |  |  |  |  |  |  |  |  |
| **Time to level completion** | **Improved** | **Mean** | 139.15 | 140.62 | 117.37 | 108.90 | 94.26 | 88.82 | 95.30 | 95.35 | 65.21 |
|  |  | **SD** | 64.16 | 75.85 | 60.71 | 49.93 | 35.87 | 28.08 | 46.74 | 40.79 | 27.39 |
|  | **Not improved** | **Mean** | 172.76 | 149.55 | 126.41 | 157.60 | 131.46 | 134.21 | 125.19 | 115.21 | 110.06 |
|  |  | **SD** | 53.97 | 53.70 | 41.24 | 62.22 | 57.35 | 39.40 | 44.38 | 63.68 | 41.80 |
|  |  |  |  |  |  |  |  |  |  |  |  |
| **Navigation efficiency** | **Improved** | **Mean** | 0.54 | 0.49 | 0.52 | 0.55 | 0.60 | 0.59 | 0.59 | 0.57 | 0.70 |
|  |  | **SD** | 0.14 | 0.22 | 0.19 | 0.15 | 0.19 | 0.18 | 0.14 | 0.14 | 0.16 |
|  | **Not improved** | **Mean** | 0.40 | 0.43 | 0.46 | 0.40 | 0.45 | 0.42 | 0.43 | 0.52 | 0.47 |
|  |  | **SD** | 0.12 | 0.19 | 0.14 | 0.18 | 0.17 | 0.13 | 0.14 | 0.17 | 0.17 |

| **Session 4** | | | **Session 5** | | | **Session 6** | | | **Session 7** | | |
| --- | --- | --- | --- | --- | --- | --- | --- | --- | --- | --- | --- |
| **Level 1** | **Level 2** | **Level 3** | **Level 1** | **Level 2** | **Level 3** | **Level 1** | **Level 2** | **Level 3** | **Level 1** | **Level 2** | **Level 3** |
| 2.10 | 2.09 | 1.85 | 2.07 | 1.89 | 1.69 | 2.09 | 2.02 | 1.82 | 1.97 | 1.91 | 1.70 |
| 1.11 | 0.83 | 0.72 | 0.88 | 0.80 | 0.63 | 0.96 | 0.98 | 1.00 | 0.75 | 0.84 | 0.67 |
| 2.18 | 2.16 | 2.08 | 2.12 | 2.04 | 1.96 | 1.95 | 1.96 | 1.89 | 1.91 | 1.80 | 1.96 |
| 0.64 | 0.70 | 0.59 | 0.56 | 0.56 | 0.58 | 0.43 | 0.72 | 0.55 | 0.50 | 0.58 | 0.86 |
|  |  |  |  |  |  |  |  |  |  |  |  |
| 25.27 | 21.52 | 17.36 | 30.77 | 20.39 | 18.77 | 29.66 | 27.86 | 23.91 | 36.11 | 24.86 | 24.05 |
| 23.13 | 10.04 | 9.89 | 18.27 | 8.34 | 8.60 | 15.57 | 17.34 | 22.39 | 18.34 | 12.44 | 11.88 |
| 40.37 | 33.34 | 36.02 | 36.00 | 32.29 | 39.26 | 39.13 | 36.26 | 37.05 | 36.03 | 32.11 | 33.61 |
| 17.79 | 11.94 | 19.32 | 13.45 | 20.59 | 12.20 | 25.42 | 16.55 | 15.49 | 14.02 | 12.00 | 20.43 |
|  |  |  |  |  |  |  |  |  |  |  |  |
| 92.77 | 79.06 | 62.28 | 106.51 | 78.02 | 68.87 | 103.64 | 90.49 | 83.50 | 110.78 | 78.23 | 73.32 |
| 91.71 | 39.71 | 24.39 | 70.58 | 56.77 | 38.38 | 56.28 | 54.85 | 92.16 | 56.23 | 35.08 | 27.55 |
| 128.94 | 101.63 | 110.05 | 111.75 | 101.64 | 116.16 | 118.93 | 113.88 | 117.14 | 107.16 | 93.54 | 95.49 |
| 62.21 | 33.09 | 52.67 | 41.18 | 71.15 | 28.83 | 78.25 | 66.14 | 60.51 | 54.88 | 37.03 | 65.24 |
|  |  |  |  |  |  |  |  |  |  |  |  |
| 0.71 | 0.72 | 0.79 | 0.70 | 0.77 | 0.76 | 0.69 | 0.73 | 0.78 | 0.64 | 0.70 | 0.73 |
| 0.16 | 0.17 | 0.10 | 0.16 | 0.14 | 0.13 | 0.16 | 0.16 | 0.13 | 0.19 | 0.19 | 0.19 |
| 0.45 | 0.48 | 0.48 | 0.45 | 0.50 | 0.46 | 0.50 | 0.50 | 0.52 | 0.47 | 0.54 | 0.54 |
| 0.19 | 0.16 | 0.21 | 0.13 | 0.17 | 0.14 | 0.16 | 0.16 | 0.17 | 0.14 | 0.17 | 0.12 |

| **Session 8** | | | **Session 9** | | | **Session 10** | | |
| --- | --- | --- | --- | --- | --- | --- | --- | --- |
| **Level 1** | **Level 2** | **Level 3** | **Level 1** | **Level 2** | **Level 3** | **Level 1** | **Level 2** | **Level 3** |
| 1.90 | 1.71 | 1.77 | 1.81 | 1.70 | 1.56 | 1.83 | 1.75 | 1.54 |
| 0.89 | 0.78 | 0.80 | 0.61 | 0.57 | 0.57 | 0.68 | 0.66 | 0.55 |
| 1.93 | 1.90 | 1.82 | 1.81 | 1.75 | 1.79 | 1.73 | 1.70 | 1.94 |
| 0.59 | 0.56 | 0.55 | 0.67 | 0.46 | 0.58 | 0.46 | 0.51 | 0.68 |
|  |  |  |  |  |  |  |  |  |
| 42.14 | 30.64 | 28.02 | 41.86 | 44.07 | 31.61 | 43.05 | 32.14 | 33.95 |
| 22.28 | 25.05 | 19.81 | 23.05 | 31.04 | 14.81 | 20.22 | 12.87 | 16.06 |
| 38.13 | 30.53 | 31.03 | 31.58 | 38.39 | 30.32 | 43.55 | 34.03 | 35.73 |
| 13.74 | 9.67 | 17.99 | 13.64 | 21.77 | 15.09 | 44.73 | 26.02 | 20.76 |
|  |  |  |  |  |  |  |  |  |
| 110.43 | 85.30 | 84.31 | 94.53 | 99.83 | 74.55 | 91.89 | 78.71 | 78.80 |
| 48.34 | 71.42 | 64.24 | 48.41 | 64.15 | 34.65 | 34.73 | 46.83 | 40.13 |
| 111.31 | 94.74 | 98.09 | 91.59 | 111.58 | 91.54 | 115.34 | 97.63 | 100.14 |
| 36.58 | 34.50 | 61.81 | 44.09 | 64.05 | 38.05 | 101.36 | 49.99 | 47.47 |
|  |  |  |  |  |  |  |  |  |
| 0.66 | 0.76 | 0.78 | 0.69 | 0.71 | 0.76 | 0.73 | 0.75 | 0.76 |
| 0.17 | 0.20 | 0.17 | 0.19 | 0.20 | 0.19 | 0.14 | 0.18 | 0.21 |
| 0.44 | 0.51 | 0.51 | 0.55 | 0.52 | 0.52 | 0.48 | 0.54 | 0.53 |
| 0.14 | 0.13 | 0.17 | 0.14 | 0.17 | 0.14 | 0.16 | 0.14 | 0.12 |

**Supplemental Table 3:** Mixed factorial ANCOVA analyses of game performance measures across group (3: CityQuest; Spatial Navigation-only; Obstacles-only), level (3) and session (10) across game training.

| **Game performance analysis** |
| --- |
| **Stop time at decision points** |
| Main effect of training group [*F*(2, 51) = 12.79, *p* < 0.001, η_p_^2^ = 0.33], with the Obstacles-only group spending significantly less time at decision points (*M* = 1.44, *SD* = 0.56) compared to the CityQuest (*M* = 2.18, *SD* = 0.5, p = 0.002) or Spatial Navigation-only groups (*M* = 2.08, *SD* = 0.71, *p* = 0.007), with no difference between these latter groups. This finding indicates that deciding the direction to pursue at an intersection was more difficult for the CityQuest and Spatial Navigation groups, compared to the Obstacles group. (See Supplemental Table 1 for mean game performance accuracy of the training groups). |
| **Number of intersections traversed** |
| Main effect of training group [*F*(2, 51) = 19.53, *p* < 0.001, η_p_^2^ = 0.43], with the Obstacles-only group traversing significantly more intersections (*M* = 43.55, *SD* = 4.10) than the CityQuest group (*M* = 32.7, *SD* = 5.78, *p* < 0.001) and the Spatial Navigation-only group (*M* = 31.79, *SD* = 7.44, *p* < 0.001). This finding is likely due to the Obstacle-only group’s task of collecting as many gems as possible in the cityscape within a time limit. No other significant main effects or interactions were found for this measure (all *p*s > 0.1). |
| **Time to level completion** |
| Main effect of training group [*F*(1, 37) = 10.18, *p* = 0.003, η_p_^2^ = 0.22], with the CityQuest group (*M* = 108.06, *SD* = 21.67) taking longer to complete each level compared to the Spatial Navigation-only group (*M* = 97.41, *SD* = 25.51). |
| **Navigation efficiency** |
| No main effect of training group [*F*(1, 37) = 2.64, *p* = 0.11, η_p_^2^ = 0.07], suggesting comparable navigation efficiency across groups. No other significant main effects or interactions were found for this measure (all *p*s > 0.065). These results suggest that navigation efficiency remained constant across levels and sessions. |
| **Obstacle avoidance efficiency** |
| Main effect of training group [*F*(1, 32) = 15.06, *p* < 0.001, η_p_^2^ = 0.32], with greater efficiency by the Obstacles-only group (*M* = 0.83, *SD* = 0.04) than the CityQuest group (*M* = 0.8, *SD* = 0.05). There was a significant interaction between training group and game level [*F*(2, 31) = 17.69, *p* < 0.001, η_p_^2^ = 0.53], which was driven by the greater obstacle avoidance efficiency of the Obstacles-only group at Level 2 compared to the CityQuest group. No other significant main effects or interactions were found (all *p*s > 0.27). |

**Supplemental Table 4**: Mean age profile and cognitive characteristics of those who a) improved and b) did not improve in both the CityQuest and Spatial Navigation-only training conditions training (with standard deviations in parentheses).

|  | Participants who did improve (N = 22) | Participants who did not improve (N = 19) | *t*-test value | *p* value |
| --- | --- | --- | --- | --- |
| Age (years) | 71.06 (3.81) | 71.22 (4.65) | 0.12 | 0.908 |
| MoCA score | 28.32 (1.67) | 25.89 (1.73) | 4.56 | 0.001* |
| SBSOD rating | 4.78 (0.90) | 4.50 (1.12) | 0.88 | 0.384 |
| Visual Acuity (LogMAR) | 0.10 (0.11) | 0.09 (0.12) | 0.42 | 0.675 |
| Contrast Sensitivity (logCS) | 1.94 (0.03) | 1.91 (0.12) | 1.22 | 0.229 |
| Hearing Acuity (dB) | 35.63 (18.42) | 30.19 (10.73) | 0.96 | 0.345 |

*significant at *p* < 0.05

The characteristics of the participants whose performance improved with training compared to those who did not are summarized in Supplemental Table 4. Both the successfully trained (9 CityQuest, 13 Spatial Navigation-only) and unsuccessfully trained (12 CityQuest, 7 Spatial Navigation-only) participants were matched in age, visual function, hearing ability, and sense of direction. However, those who improved with training had a significantly higher MoCA score than those who did not (see Supplemental Table 4), although all participants scored above the recommended cut-off (Luis et al., 2009). It is important to note, however, that the MoCA is used as a screening tool for cognitive impairment and comparisons of scores above this cut-off point may not always be appropriate as the diagnostic test accuracy of the tool decreases with higher scores (Luis et al., 2009; Quinn et al., 2021). As such, we did not include a MoCA score as a covariate in subsequent analyses.

**Spatial Navigation Assessment**

We conducted a mixed ANOVA with Improvement group (3: training-improved; training-no improvement; control (i.e. Obstacles-only)) and time (2: pre-, post-training) on performance accuracy to the spatial navigation assessment. See Supplemental Table 5 for mean performance accuracy of the improvement group across each of the pre- and post-training assessments.

For the object recognition task, there was a main effect of improvement group [*F*(2, 53) = 4.96, *p* = 0.011, η_p_^2^ = 0.16], with greater accuracy scores for the Improved group (*M* = 90.63, *SD* = 6.1) compared to the No improvement group (*M* = 83.22, *SD* = 8.79, *p* = 0.008), whose performance did not differ from that of the Obstacles-only group (*M* = 88.33, *SD* = 7.96, *p* = n.s.). There was an effect of time [*F*(1, 53) = 7.27, *p* = 0.009, η_p_^2^ = 0.12], with better performance post- (*M* = 89.84, *SD* = 10.5) than pre- training (*M* = 85.16, *SD* = 10.83, *p* = 0.014). There was no interaction between improvement group and time [*F*(2, 53) = 1.65, *p* = 0.2, η_p_^2^ = 0.06].

The analysis of performance to the direction judgement task showed a main effect of improvement group [*F*(2, 53) = 5.96, *p* = 0.005, η_p_^2^ = 0.18] with highest accuracy scores for the Improved group (*M* = 76.99, *SD* = 12.99) compared to the No improvement group (*M* = 62.5, *SD* = 15.02, *p* = 0.006), whose performance did not differ from that of the Obstacles-only group (*M* = 65, *SD* = 15.27, *p* = 0.041). There was no effect of time, nor any evidence of interactions between these factors [all *F* ratios < 1].

The analysis of the accuracy performance to the landmark location task revealed a main effect of Improvement group [*F*(2, 53) = 8.53, *p* = 0.001, η_p_^2^ = 0.24], with highest accuracy scores for the Improved group (*M* = 73.72, *SD* = 15.18) compared to either the No improvement group (*M* = 50.16, *SD* = 19.39, *p* < 0.001), or the Obstacles-only group (*M* = 58.75, *SD* = 21.59, *p* = 0.049). There was an effect of time [*F*(1, 53) = 9.18, *p* = 0.004, η_p_^2^ = 0.15], with performance improving from pre- (*M* = 56.47, *SD* = 22.42) to post-training (*M* = 66.96, *SD* = 25.47, *p* = 0.002). There was no interaction between group and time [*F*(1, 52) < 1].

The mixed ANOVA on performance in the landmark naming task showed a main effect of Improvement group [*F*(2, 53) = 8.26, *p* = 0.001, η_p_^2^ = 0.24], with highest accuracy scores for the Improved group (*M* = 77.27, *SD* = 22.21) compared to the No improvement group (*M* = 50.99, *SD* = 19.46, *p* < 0.001). There was no difference in accuracy between the Obstacles-only group (*M* = 62.92, *SD* = 19.97) and the other two groups (all *p*s > 0.11). There was no effect of time [*F*(1, 53) < 1] and no interaction between these factors [*F*(2, 53) < 1].

**
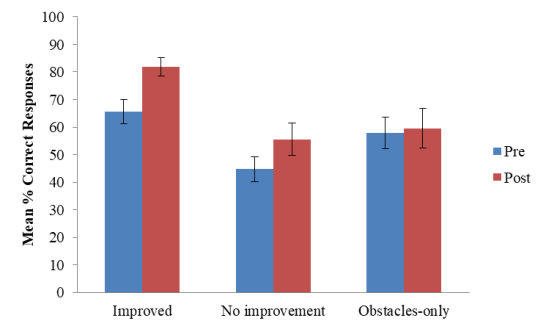
**

**Supplemental Figure 1**: Mean percentage accuracy on the landmark location task across assessments (pre and post) for those who improved in spatial navigation training, those with no improvement, and the control group (Obstacles-only). Error bars indicate ± 1 standard error of the mean.

**Spatial Strategy Assessment**

We then compared performance on the spatial strategy assessment for participants who successfully trained with those who did not train successfully or were allocated to the obstacle avoidance training, using a series of mixed ANOVAs.

The analysis of variance of performance to the ‘same direction’ trials revealed no effect of group [*F*(2, 53) < 1]. There was a main effect of time [*F*(1, 53) = 4.53, *p* = 0.038, η_p_^2^ = 0.08], with performance improving from pre- (*M* = 78.57, *SD* = 17.43) to post-assessment (*M* = 83.48, *SD* = 15.1, *p* = 0.033). There was no interaction between training group and time [*F*(2, 53) < 1].

For the ‘different direction’ trials, performance across the Improved group, No improvement group, and obstacle avoidance group was not significantly better than chance at either pre-training or post-training [all *t* values < 1], with the exception of the performance of the Improved group at post-training [*t*(21) = 2.86, *p* = 0.009]. The mixed ANOVA showed no effect of Improvement group [*F*(2, 53) < 1], no effect of time [*F*(1, 53) = 3.8, *p* = 0.056, η_p_^2^ = 0.07], and no interaction between these factors [*F*(2, 53) < 1].

We then calculated, for each participant, the number of responses in the ‘different trials’ which were in line with an associative cue, beacon, and allocentric navigation strategy at pre- and post-training. We conducted a mixed ANOVA with Improvement group at training (3; Improved, no improvement, and obstacle avoidance group) as the between group factor, and time (2: pre-, post-training) and strategy type (3: associative cue, beacon, allocentric) as the within group factors. Analysis showed no effect of improvement group [*F*(2, 53) = 1.81, *p* = 0.174, η_p_^2^ = 0.06] or time [*F*(1, 52) < 1]. There was a main effect of strategy use [*F*(2, 52) = 64.38, *p* < 0.001, η_p_^2^ = 0.7] with a post-hoc analysis revealing a preference for associative cue (*M* = 46.43, *SD* = 11.82) and beacon strategies (*M* = 40.18, *SD* = 11.50) over an allocentric strategy (*M* = 12.28, *SD* = 13.49). There was no interaction between group and time [*F*(2, 53) < 1], no interaction between group and strategy [*F*(4, 102) < 1], nor any interaction between group, time, and navigation strategy [*F*(4, 102) = 1.04, *p* = 0.39, η_p_^2^ = 0.04]. Thus, there was no evidence that the intervention led to a shift in the use of navigation strategy in either groups.

**Executive function**

We compared TMT performance between those whose performance improved during the spatial navigation training with those whose performance did not improve during training and those allocated to the Obstacles-only group using a mixed ANOVA. The analysis revealed no effect of improvement group [*F*(2, 51) = 2.58, *p* = 0.086, η_p_^2^ = 0.09] but a main effect of time [*F*(1, 51) = 12.77, *p* = 0.001, η_p_^2^ = 0.2] with performance improving following training. There was no interaction between improvement group and time [*F*(2, 51) = 1.4, *p* = 0.25, η_p_^2^ = 0.05].

**Supplemental Table 5**: Mean performance accuracy across the spatial navigation and spatial strategy assessments, percentage strategy preference, and completion times for the trail making test at pre- and post-training for those who improved in spatial navigation training and for those who did not improve (with standard deviations in parentheses).

|  | **Pre-Training** | | | | **Post-Training** | |
| --- | --- | --- | --- | --- | --- | --- |
|  | **Spatial Navigation Assessment** | | | | | |
|  | **Improved** | **No improvement** | **Improved** | | | **No improvement** |
|  | N = 22 | N = 19 | N = 22 | | | N = 19 |
| **Object Recognition** | 88.07 (10.72) | 82.89 (10.17) | 93.18 (7.93) | | | 83.55 (12.19) |
| **Direction Judgement** | 74.43 (18.29) | 61.18 (17.63) | 79.55 (16.16) | | | 63.82 (21.61) |
| **Landmark Location** | 65.63 (20.75) | 44.74 (20.12) | 81.82 (15.42) | | | 55.59 (25.59) |
| **Landmark Naming** | 73.30 (25.96) | 51.97 (24.39) | 81.25 (22.41) | | | 50.00 (25.69) |
|  |  |  |  | | |  |
|  | **Spatial Strategy Assessment** | | | | | |
| **Same Direction** | 82.20 (15.17) | 76.10 (21.19) | 86.36 (12.28) | | | 82.02 (19.15) |
| **Different Direction** | 32.48 (11.26) | 33.77 (13.62) | 39.68 (10.96) | | | 35.53 (13.53) |
|  |  |  |  | | |  |
|  | **Strategy Preference** | | | | | |
| **Associative** | 47.35 (16.54) | 46.49 (16.03) | 44.32 (15.72) | | | 51.32 (18.89) |
| **Beacon** | 40.91 (14.75) | 43.86 (11.40) | 35.98 (21.11) | | | 40.35 (16.02) |
| **Allocentric** | 10.61 (17.29) | 9.65 (12.19) | 17.80 (19.64) | | | 7.89 (12.26) |
|  |  |  |  | | |  |
|  | **Trail Making Test** | | | | | |
| **Completion Time** | 47.26 (21.63) | 71.20 (41.78) | | 36.46 (18.06) | | 48.02 (36.67) |
|  |  |  | |  | |  |

**Supplemental Table 6:** Mixed factorial ANOVA analyses of game performance measures across group (3: spatial navigation training - improved; spatial navigation training - no improvement; no spatial navigation training - Obstacles-only), level (3) and session (10) across game training.

| **Game performance analysis** |
| --- |
| **Stop time at decision points** |
| Main effect of group [*F*(2, 53) = 7.36, *p* = 0.002, η_p_^2^ = 0.22]. Obstacles-only (control) group spent significantly less time at decision points (*M* = 1.44, *SD* = 0.56) compared to the Improved group (*M* = 2.11, *SD* = 0.7, *p* = 0.004) and the No improvement group (*M* = 2.16, *SD* = 0.49, *p* = 0.003). Possibly due to the non-relevance of decision points for the Obstacles-only group. (See Supplemental Table 2 for mean game performance accuracy across Improvement groups). |
| Main effect of session [*F*(9, 45) = 14.55, *p* < 0.001, η_p_^2^ = 0.74]. This was driven by participants’ mean stop time decreasing significantly across sessions e.g., from Session 1 (*M* = 3.02, *SD* = 1.17) to Session 10 (*M* = 1.55, *SD* = 0.63). |
| Main effect of level [*F*(2, 52) = 14.06, *p* < 0.001, η_p_^2^ = 0.35]. Significant interaction between group and level [*F*(4, 104) = 4.11, *p* = 0.004, η_p_^2^ = 0.14]. This interaction was driven by the control group spending less time at decision points across levels compared to the Improved group and No improvement group (all *p*s < 0.011). |
| **Number of intersections traversed** |
| Main effect of group [*F*(2, 53) = 33.82, *p* < 0.001, η_p_^2^= 0.56]. Obstacles-only group traversed the most intersections (*M* = 43.55, *SD* = 4.1), followed by the No improvement group (*M* = 35.97, *SD* = 4.57) and the Improver group travelled the least amount of intersections (*M* = 29.06, *SD* = 6.42) (all *p*s < 0.001). |
| Main effect of level [*F*(2, 52) = 55.47, *p* < 0.001, η_p_^2^ = 0.68]. Significant interaction between group and level [*F*(4, 104) = 3.68, *p* = 0.008, η_p_^2^ = 0.12]. This interaction was driven by the Improved group traveling through fewer intersections from Level 1 to 3, than both the No improvement group and the control group (all *p*s < 0.001). This was possibly due to the performance of the Improved group becoming more efficient at locating target locations across levels, thus not needing to travel through as many intersections. |
| Significant interaction between group and session [*F*(18, 90) = 1.8, *p* = 0.038, η_p_^2^ = 0.26]. This interaction was driven by the Improved group traveling through fewer intersections from Session 2 to 7, than both the No improvement group and the control group (all *p*s < 0.001). |
| **Time to level completion** |
| Main effect of group [*F*(1, 39) = 11.55, *p* = 0.001, η_p_^2^ = 0.23]. The Improver group took significantly less time to complete each level of game training (*M* = 92.36, *SD* = 23.34) compared to the No improvement group (*M* = 115.02, *SD* = 18.62, *p* = 0.002). |
| Main effect of session [*F*(9, 31) = 6.26, *p* < 0.001, η_p_^2^ = 0.65]. This was driven by participants taking longer to complete Session 1 compared to all other sessions, with the exception of Session 2 (all *p*s < 0.001). |
| Main effect of level [*F*(2, 38) = 24.69, *p* < 0.001, η_p_^2^ = 0.57]. Significant interaction between group and level [*F*(2, 38) = 3.52, *p* = 0.04, η_p_^2^= 0.16]. This was driven by Improved group completing each Level 3 significantly faster than the No improvement group (*p* < 0.001). |
| **Navigation efficiency** |
| Main effect of group [*F*(1, 39) = 65.84, *p* < 0.001, η_p_^2^ = 0.63]. The Improved group was more efficient at navigating (*M* = 0.68, *SD* = 0.09) compared to the No improvement group (*M* = 0.48, *SD* = 0.05). |
| Main effect of session [*F*(9, 31) = 9.68, *p* < 0.001, η_p_^2^ = 0.74]. Significant interaction between group and session [*F*(9, 31) = 2.54, *p* = 0.026, η_p_^2^ = 0.43]. This was driven by better navigation efficiency from Session 2 to 10 by the Improved group compared to the No improvement group (all *p*s < 0.001). |
| Main effect of level [*F*(2, 38) = 18.34, *p* < 0.001, η_p_^2^ = 0.49]. Significant interaction between group and level [*F*(2, 38) = 3.34, *p* = 0.046, η_p_^2^ = 0.15]. This interaction was driven by the Improved group navigating more efficiently at Levels 1, 2, and 3 compared to the No improvement group (all *p*s < 0.001). |

**References**

Luis, C. A., Keegan, A. P., & Mullan, M. (2009). Cross validation of the Montreal Cognitive Assessment in community dwelling older adults residing in the Southeastern US. *International Journal of Geriatric Psychiatry*, *24*(2), 197–201. https://doi.org/10.1002/gps

Quinn, T. J., Richard, E., Teuschl, Y., Gattringer, T., Hafdi, M., O’Brien, J. T., Merriman, N., Gillebert, C., Huyglier, H., Verdelho, A., Schmidt, R., Ghaziani, E., Forchammer, H., Pendlebury, S. T., Bruffaerts, R., Mijajlovic, M., Drozdowska, B. A., Ball, E., & Markus, H. S. (2021). European Stroke Organisation and European Academy of Neurology joint guidelines on post-stroke cognitive impairment. In *European Stroke Journal* (Vol. 6, Issue 3). https://doi.org/10.1177/23969873211042192
